# Deep learning-guided structural analysis of a novel bacteriophage KPP105 against multidrug-resistant *Klebsiella pneumoniae*

**DOI:** 10.1016/j.csbj.2025.04.032

**Published:** 2025-05-01

**Authors:** Seyoung Ko, Jaehyung Kim, Jae-Hyun Cho, Youngju Kim, Donghyuk Kim

**Affiliations:** a School of Energy and Chemical Engineering, Ulsan National Institute of Science and Technology (UNIST), Ulsan 44919, Republic of Korea; b Optipharm Inc., Cheongju-si, Chungcheongbuk-do, Republic of Korea

**Keywords:** *Klebsiella pneumoniae*, Bacteriophage, Genomic analysis, Protein structure prediction, Structural analysis

## Abstract

The increasing prevalence of multidrug-resistant bacteria, particularly *Klebsiella* species, poses a significant global health threat. Bacteriophages have emerged as promising alternatives due to their specificity and efficacy against bacterial targets. Characterizing phages, alongside analyzing their protein structures provide crucial insights into their host specificity, infection mechanisms, and potential applications. In this study, we isolated a novel bacteriophage, KPP105, and conducted comprehensive physiological, genomic, and structural analysis. Physiological assessments revealed that KPP105 maintains stable activity across a wide range of pHs and temperature conditions and exhibits host-specific infection properties. Genomic analysis classified KPP105 as a member of the *Demerecviridae* family and identified it as a lytic bacteriophage harboring a lytic cassette. Deep learning-based structural analysis of host-interacting proteins, including the receptor-binding protein (RBP) and endolysin derived from KPP105, was performed. Structural similarity analysis indicated that its RBP facilitates interactions with host receptors and exhibits unique sequence patterns distinguishing *Klebsiella* strains from other bacteria. Structure-based functional analysis provided comprehensive insights into cell wall degradation with various peptidoglycan fragments. In conclusion, this study reports the physiological, genomic, and structural characteristics of the novel lytic bacteriophage KPP105, offering valuable insights into its potential as an alternative agent against multidrug-resistant *Klebsiella* infections.

## Graphical Abstract

**Figure:**
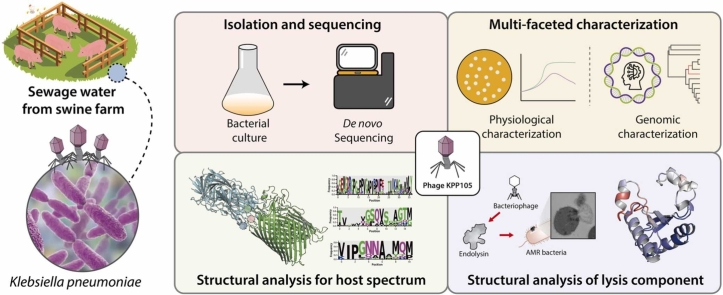

## 1 Introduction

*Klebsiella pneumoniae* is an opportunistic Gram-negative pathogen that presents significant clinical challenges due to its ability to acquire antimicrobial resistance determinants [1] and form biofilms [2], [3], which contribute to its resistance to standard treatment methods. In particular, the growing prevalence of drug-resistant strains of *K. pneumoniae*, including those resistant to multiple drugs such as carbapenems, has become a significant global health concern [4], [5], [6], [7]. In response, the Centers for Disease Control and Prevention (CDC) began active surveillance of carbapenemase-producing *K. pneumoniae* in 2009 as part of its control guidelines. Furthermore, in 2017, the World Health Organization (WHO) designated *K. pneumoniae* as a critical priority pathogen, emphasizing the urgent need for new therapeutic solutions [8]. The emergence of multidrug-resistant *K. pneumoniae* therefore represents a global public health crisis, highlighting the need for alternative treatment options.

Bacteriophages have emerged as promising candidates for controlling infections caused by drug-resistant pathogens due to their ability to selectively target and lyse specific bacteria [9], [10]. This specificity sets bacteriophages apart from broad-spectrum antibiotics, which can disrupt natural microbial communities and cause collateral damage to beneficial bacteria [11]. The therapeutic potential of bacteriophages has been demonstrated in both *in vitro* and *in vivo* studies [12], [13], [14], [15]. In particular, endolysins, a phage-derived lytic protein, have gained attention as a potential antimicrobial agent due to their ability to cleave the peptidoglycan (PG) layers of bacterial cell walls. Previous studies [16, [17], [18], [19, [20], [21] have shown that the exogenous application of endolysins efficiently degrades PG layers and exhibits antimicrobial activity. However, its efficacy against Gram-negative bacteria is limited by the structural sophistication of their cell walls, which impedes lytic activity. To overcome this limitation, alternative strategies have been explored to enhance membrane permeability by using destabilizing agents such as EDTA [22], [23], [24], [25], [26]. Further studies are required to optimize the lytic activity of endolysins through various engineering approaches. In this context, a comprehensive understanding of the structural and functional characteristics of phage-derived proteins serves as a crucial basis for exploring their diverse applications, including antimicrobial strategies. This includes not only endolysins but also other phage-derived proteins, such as receptor-binding proteins (RBPs), which are critical for host recognition and infection. However, the structural characterization of these proteins has traditionally relied on experimental methods, which are time-consuming and resource-intensive, thereby hindering progress in the field.

Recent advancements in deep learning-based structure prediction technologies [27], [28], such as AlphaFold, have introduced a groundbreaking approach for accurately predicting protein structures that significantly reduces the reliance on experimental validation. These tools can generate three-dimensional structures directly from protein sequences, dramatically reducing time and resource requirements while enabling a wide range of structure-based analyses [9], [29], [30]. This progress has facilitated the generation of large-scale structural datasets in a high-throughput manner, supporting numerous biological studies. Previous research has applied these technologies to investigate bacteriophage-derived proteins, such as endolysins and RBPs, uncovering their functional mechanisms [31], [32], [33]. Furthermore, methods for efficiently analyzing protein networks based on structural similarities [34] have been developed, allowing for quantitative evaluations of structural correlations and the discovery of novel functional relationships within extensive biological datasets [35]. These advancements underscore the critical role of deep learning-based structure prediction in elucidating the intricately complex relationship between protein structure and function. Moreover, these technologies go beyond facilitating physiological and bioinformatics-based analysis by enabling large-scale functional predictions through the use of high-throughput data, thereby significantly enhancing the efficiency of bacteriophage research.

Herein, this study presents a comprehensive approach to investigate the characteristics of the newly isolated bacteriophage KPP105. The novel bacteriophage, which specifically targets and lyses strains of *K. pneumoniae*, was assessed using both physiological and bioinformatics-based analysis. Additionally, cutting-edge protein structure prediction techniques were employed to predict and examine the three-dimensional structures of phage-derived proteins. These approaches provide new insights into the structural features and functional mechanisms of these proteins, emphasizing the potential for more detailed assessments of phage-derived proteins. The findings highlight the promising applications of bacteriophage in combating drug-resistant pathogens and provide essential data for future research on protein-based therapies and related applications.

## 2 Materials and methods

### 2.1 Host bacteria and culture conditions

To isolate bacteriophage that target *K. pneumoniae* producing extended-spectrum beta-lactamase (ESBL (+) *K. pneumoniae*), the #KPN10 strain (ID: KBN K-1) was used as the host bacteria and grown in Tryptic Soy Broth (TSB, BD, Germany) at 35 °C for 18 hours. Other bacterial strains examined in this research were cultivated in TSB at 35 °C for the same duration.

### 2.2 Isolation and purification of bacteriophage

The bacteriophage samples were collected from sewage near a swine farm in Daejeon, South Korea [36]. The samples were homogenized and filtered through a membrane filter with 0.2 μm pore size (Sartorius, Germany). The mixture of 18 mL filtrate, 2 mL of 10X TSB, and 300 μL of host bacterial culture was incubated at 35 °C for 18 hours. Following centrifugation at 4000 rpm for 20 min, the supernatant was filtered through a 0.45 μm membrane filter. A 10 μL sample of the filtrate was spotted onto the surface of a Tryptic Soy Agar (TSA) plate that had been overlaid with 0.6 % TSB soft agar containing cultured host cells of *K. pneumoniae* strain KBN K-1. The plate was incubated at 35 °C for 24 hours, after which zones of lysis were observed. This assay, based on the soft agar overlay method, was used for the initial detection of phage lytic activity.

Soft agar overlay method [37] was performed to purify the bacteriophage from the culture medium. The supernatant obtained after initial enrichment and filtration was diluted with SM buffer (100 mM NaCl, 10 mM MgSO4, 50 mM Tris-HCl with pH 7.5) by 10-fold. Diluted sample (100 μL), host bacterial culture (150 μL), and 0.6 % TSB top agar (3 mL) were mixed and spread onto a TSA plate with 2 % agar. This was then incubated for 24 hours at 35 °C. The single plaque was selected and resuspended in 400 μL of SM buffer and layered at room temperature for 4 hours. The above suspension of 100 µL was mixed with 12 mL of 0.6 % TSB top agar and 300 μL of host bacteria culture. The mixture was then cultured for 24 hours at 35 °C. To extract bacteriophage, 15 mL of SM buffer was poured onto the cultured plate and was stirred slowly at room temperature for 4 hours. The suspension was recovered and was centrifuged at 4000 rpm for 20 minutes at 4 °C. For the concentration and purification of bacteriophage lysates, procedures involving polyethylene glycol 8000 (PEG 8000, Sigma-Aldrich, United States) precipitation and cesium chloride (CsCl, Sigma-Aldrich, United States) gradient ultracentrifugation were performed. The supernatant, which contained the phages, was then subjected to precipitation by gradually incorporating PEG 8000 until it reached a final concentration of 10 % w/v. This mixture was stirred at 4 °C overnight. After centrifugation at 7200 ×g, 4 °C for 20 minutes yielded PEG-bacteriophage pellets, which were cleansed twice using 0.1 M ammonium acetate (pH 7.0). These pellets were then resuspended in 0.5 mL of sterile distilled water and stored at 4 °C overnight prior to the further purification step. The bacteriophage suspension was subsequently refined through CsCl gradient ultracentrifugation. This involved introducing the bacteriophage solution into a CsCl gradient with densities of p = 1.70, p = 1.50, p = 1.45, and p = 1.30, and then centrifuging the mixture at 78,500 ×g at 4 °C for 2 hours. The light-gray band that formed was collected and dialyzed three times using the membrane (SPECTRA/Pro 4 Dialysis membrane, United States) using 500 mL of SM buffer at 4 °C. The purified bacteriophage suspension was stored at 4 °C for subsequent experimentation.

### 2.3 One-step growth curve analysis of bacteriophage

The one-step growth curve analysis to determine the latent time and burst size was conducted as described previously [38], with some modifications. Briefly, 10 μL of bacteriophage suspension (approximately 1.0 × 10^9^ PFU/mL) was added to 10 mL of exponential phase culture of *K. pneumoniae* (1.0 × 10^8^ CFU/mL) to obtain the multiplicity of infection (MOI) of 0.01. The bacteriophage was allowed to adsorb onto the bacterial surface for 5 minutes at room temperature. Subsequently, the mixture was centrifuged at 10,000 ×g for 1 minute and the resultant pellet was resuspended in 20 mL of TSB medium. Aliquots were collected at 5-minute intervals, and the bacteriophage titer was measured with the standard double-layer top agar assay [39].

### 2.4 Host-range determination for bacteriophage

The host range of the isolated bacteriophage KPP105 was tested against 15 ESBL (+) *K. pneumoniae* strains and 9 other Gram-negative bacteria. The ESBL (+) *K. pneumoniae* were obtained from Asian Bacterial Bank (ABB) of Asia Pacific Foundation for Infectious Diseases (APFID, Seoul, South Korea), which were all isolated from human blood. The other Gram-negative bacteria were *Acinetobacter baumannii* ATCC17978, *Citrobacter freundii* clinical isolate 15–0628, *Cronobacter sakazakii* KCTC 2949, *Escherichia coli* KCTC 1039, ESBL (+) *Escherichia coli* K01-ECO12–052, *Proteus mirabilis* KCTC2566, *Pseudomonas aeruginosa* KCTC2004, *Salmonella Typhimurium* ATCC14028, and *Salmonella Enteritidis* KCCM12021. All of these bacteria were handled by Optipharm Inc. using the standard soft agar overlay method for the host specificity test [37]. Briefly, a fresh bacterial culture (150 μL) was mixed with 3 mL of 0.6 % TSB top agar and poured onto TSA plates. After solidification, 10 μL of the phage suspension was spotted onto the surface. The plates were incubated at 35 °C for 15 hours, after which lysis plaques were examined. Lytic activity was evaluated based on the clarity and size of the spots. Bacterial strains exhibiting clear lysis were considered susceptible to phage infection.

For strains showing positive lysis in the spot assay, the efficiency of plating (EOP) of bacteriophage was determined using the same soft agar overlay method under identical conditions [40]. Phage suspensions with equal titers (PFU/mL) were applied to all strains, and the number of resulting plaques was counted. The EOP of bacteriophage was calculated as (average PFU on target bacteria/average PFU on host bacteria) × 100. The EOP value obtained on the host strain (*K. pneumoniae* KBN K-1) was defined as 100 %. The experiment was conducted in triplicate.

#### 2.4.1 Analysis of bacteriophage proteins

Phage samples were centrifuged with CsCl (Cesium Chloride, Sigma Aldrich, USA) gradient at 26,000 rpm for 4 hours. The collected pellets were suspended with SM buffer, yielding a concentration 1.0 × 10^9^ PFU/mL. Subsequently, 10 μL of 5X loading buffer was added to 40 μL of the phage suspension and boiled at 100 °C for 10 minutes. The mixture was loaded on a 12.5 % SDS-PAGE gel and electrophoresis was performed.

### 2.5 Evaluation of pH and thermal stability

To assess the temperature stability of isolated bacteriophage KPP105, present at a concentration of 10^8^ PFU/mL, it was subjected to incubation at multiple temperatures (35, 40, 50, 60, and 70 °C) for one hour. The resulting phage titer was then determined using the soft agar overlay method. Additionally, to evaluate the pH stability of the phage KPP105, its infectivity was assessed after exposure to a range of pH conditions. Specifically, buffer solutions were prepared using acetic acid and sodium acetate buffer (pH levels 2–6), phosphate buffer (pH levels 7–11), and Tris-HCl buffer (pH levels 8–11), with pH values adjusted using 1 M HCl or 1 M NaOH. For each pH buffer (990 μL), a 10 μL phage suspension containing 1.0 × 10^9^ PFU/mL was added and gently mixed. The mixtures were incubated at room temperature for 2 hours. After incubation, the treated suspensions were subjected to phage titration using the soft agar overlay method to determine the number of remaining infectious particles.

### 2.6 Transmission electron microscopy

Glow-discharged carbon-coated copper grids were used to apply a purified bacteriophage suspension with a concentration of over 1.0 × 10^9^ PFU/mL. The sample was allowed to absorb for 2 minutes before blotting off the buffer solution onto a Whatman paper. The grids were then stained with a 2 % (w/v) uranyl acetate (UrAc) solution for 1 minute, followed by blotting off the UrAc. The results were recorded using the Tecnai G2 Spirit Twin microscope (FEI, United States) with an acceleration voltage of 120 kV.

### 2.7 Whole-genome sequencing of isolated bacteriophage

The bacteriophage suspension was treated with DNase I (TAKARA, Japan) to remove the host DNA. EDTA (20 mM), proteinase K (50 μg/mL, Qiagen), and SDS (0.5 % w/v) were added to the suspension and incubated at 50 °C for one hour. The mixture was then combined with phenol-chloroform-isoamyl alcohol (25:24:1) and centrifuged at room temperature for 10 minutes. The recovered supernatant was subjected to a second round of mix with an equal volume of phenol-chloroform-isoamyl alcohol and centrifugation. Afterwards, the supernatant was mixed with 10 % (v/v) of the total volume of 3 M sodium acetate, followed by the addition of a double volume of cold 95 % ethyl alcohol. The mixture was incubated at −20 °C for one hour and then centrifuged at 13,000 rpm for 10 minutes at 0 °C. The resulting DNA pellet was washed twice with cold 70 % ethyl alcohol. After removing the ethyl alcohol, the pellet was dried and dissolved in 100 μL TE buffer (Tris-EDTA, pH 8.0). The isolated bacteriophage KPP105 was subjected to whole genome sequencing using the illumina Hiseq2000 and PacBio RSII platform (Macrogen, Korea), respectively.

### 2.8 Genome reconstruction and sequence-based analysis of KPP105

The genome sequences of KPP105 were annotated using the RASTtk. To assign potential functions for each annotated gene, BLASTp analysis against National Center for Biotechnology Information (NCBI) non-redundant protein sequence database (NCBI-nr) was conducted with 95 % identity and 90 % coverage. The circular visualization for genome reconstruction was performed using Circos software. For identification of phage-derived lysis proteins, the protein encoding genes of KPP105 were queried against curated lysis protein sequences in PHROG databases [41] using BLASTp with defined thresholds (50 % minimum identity, 85 % minimum coverage, and 1.0E-09 minimum e-value). Potential anti-CRISPR proteins encoded in the KPP105 genome were identified using the AcrFinder [42], with the virus-specific default parameters, including a maximum intergenic distance of 250 bp. For expanded functional annotation, protein sequences assigned to the categories “moron,” “auxiliary metabolic genes,” and “host takeover” were extracted from the PHROG database. Coding sequences from the KPP105 genome were subsequently subjected to BLASTp searches against these extracted sequences to assess pairwise sequence similarity and to infer putative functions.

### 2.9 Machine learning model construction of bacteriophage family classification

To collect the complete genomic information of bacteriophages, the sequences of bacteriophages infecting eight major antibiotic-resistant bacteria including ESKAPE (*Escherichia coli*, *Salmonella* spp., *Staphylococcus aureus*, *Enterococcus* spp., *Campylobacter jejuni*, *Klebsiella pneumoniae*, *Acinetobacter baumannii*, *Pseudomonas aeruginosa*) were downloaded from NCBI Refseq database. Subsequently, the protein sequences including the “Tail” term in the selected genomic data of *Caudoviricetes* with dsDNA in the Baltimore classification scheme were used as a dataset for the family classification model [43]. The family classification model was constructed using a random forest classification algorithm, an ensemble machine learning method with 1000 estimators. Each of the collected protein sequences was represented in terms of numeric features computed from symbolic information in the protein sequence and biochemical information using ProtParam implementation of Biopython. In details, a total of thirteen physico-chemical feature descriptors (Amino acid composition, dipeptide composition, Molecular weight, Aromaticity, Instability, Isoelectric point, Grand average of hydrophobicity, Charge of neutral pH, Fraction of amino acids participating in helix, turn and sheet, Extinction coefficient of assuming reduced cysteine) were used for feature extraction. Furthermore, stratified 10-fold cross validation was employed to evaluate the feature datasets, verifying the absence of bias.

### 2.10 Pan-genome, phylogenetic analysis, and genus-level taxonomic assignment

As of November 2024, a total of 151 Reference Sequences (RefSeq) of bacteriophages with an infection host of *Klebsiella pneumoniae* have been deposited in the NCBI virus database. These sequences were downloaded for pan-genome analysis and phylogenetic analysis. The BPGA software [44], along with the USEARCH clustering tool [45] (identity threshold = 50 %, MUSCLE alignment, and UPGMA algorithm), was used to evaluate a phylogeny of 152 bacteriophages, which included KPP105. A core genome-based phylogenetic tree generated from the evaluated phylogeny was visualized through interactive Tree of Life (iTOL) v6 [46].

Since pan-genomic analysis can determine relationships between bacteriophage families, pan-genomic analysis was performed on KPP105 and its phylogenetically clustered bacteriophages. The pan-genomic analysis was conducted concomitantly with the phylogenetic analysis through BPGA software to identify the core, accessory, and unique genomes. In this context, the pan-genome refers to the complete repertoire of non-redundant genes across the analyzed phage genomes, which can be classified into the core genome (genes shared by all genomes), accessory genome (genes shared by some but not all genomes), and unique genes (genes specific to a single genome) [47], [48], [49], [50]. These classifications provided insight into gene conservation and variability among related bacteriophages and served as the basis for the phylogenetic clustering and gene composition profiles presented in Figure S3. Accordingly, the core-genome-based phylogenetic tree, together with accessory and unique gene distributions, enabled a comprehensive assessment of the evolutionary relationships and genomic features of KPP105 within its cluster.

To determine the genus-level classification of bacteriophage KPP105, comparative genome alignments were performed using representative genomes from each genus within the *Demerecviridae* family. Genus-level taxonomy was obtained from the official ICTV classification, specifically the Master Species List (MSL) version 40.v1 released in 2024, which includes 15 genera classified under the *Demerecviridae* family. Of these, two genera (*Konovirus* and *Keyvirus*) were excluded due to the absence of RefSeq-annotated genome entries in the NCBI Virus database. For the remaining 13 genera, one representative RefSeq genome was randomly selected from each genus. Along with the genome of KPP105, a total of 14 genomes were used for pairwise ANI calculation (Table S4). Average Nucleotide Identity (ANI) values were calculated using the OrthoANI tool (version 0.7.0) [51], which allows reliable comparisons even at similarity levels below 70 %. Pairwise ANI values were computed across all genome pairs, and the resulting similarity matrix was used to evaluate the taxonomic proximity of KPP105. Genus-level assignment was made based on the highest observed ANI value among the compared genera.

### 2.11 Deep learning-based structural prediction and functional analysis of proteins

The amino acid sequences of RBPs and the putative endolysin were submitted to AlphaFold3 [28] for structure prediction. AlphaFold3 was installed locally in a GPU-enabled environment to apply various ligand structures generated from Simplified Molecular Input Line Entry System (SMILES) representations which are not supported on the server. For each prediction, five models were generated with a fixed seed value of 37 to ensure reproducibility. To predict protein-ligand complex structures, five ligands were selected from the PubChem and Chemical Compounds Deep Profiling Services (CC-DPS) databases: N-acetylmuramic acid (NAM, PubChem CID 5462244), N-acetylglucosamine-N-acetylmuramic acid dimer (NAG-NAM, PubChem CID 72210857), NAM-L-alanine (NAM-L-Ala, PubChem CID 10970945), NAM-L-alanyl-D-isoglutamine (NAM-L-Ala-γ-D-Glu, PubChem CID 451714), NAM-L-Ala-γ-D-Glu-L-Lys-D-Ala-D-Ala (CC-DPS CT1103206303). The binding affinities of the five predicted models were calculated using GNINA, and the structure with the highest ranking was selected for visualization. The resulting structures were then used to evaluate potential interactions between the proteins and their ligands. Receptor-binding proteins from various predicted hosts were subjected to structural alignments using FoldMason [52]. The aligned structures were analyzed and visualized as a phylogenetic tree using the Interactive Tree of Life (iTOL) v6 [46]. For endolysin, the enzyme commission (EC) number was predicted using DeepFRI [35], and functional residues were identified through gradient-weighted class activation mapping (GradCAM) analysis. The DeepFRI model was retrained using a publicly available PDB enzyme dataset to enhance prediction accuracy, with residues identified as functionally important by GradCAM were mapped to the predicted structure, highlighting regions potentially involved in enzymatic activity.

## 3 Results

### 3.1 Characterization of newly isolated bacteriophage KPP105: morphology, growth dynamics, host specificity, and stability

**Fig. 1:**
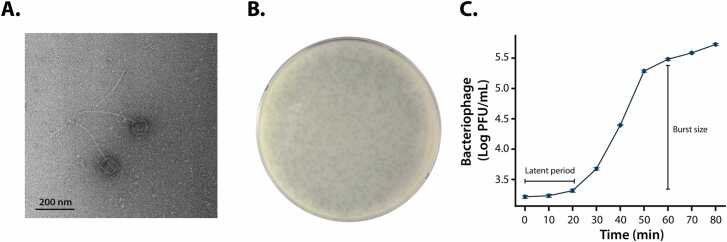
Isolated bacteriophage KPP105 infecting *Klebsiella pneumoniae*. (A) TEM micrograph showing isolated bacteriophage KPP105. (B) Plaque morphology of bacteriophage KPP105 on an agar plate. (C) One-step growth curve of KPP105, indicating a latent period of 20 minutes and a burst size of 146 PFU per infected cell.

Bacteriophage KPP105 was isolated from a sewage sample using *Klebsiella pneumoniae* KBN K-1 as an indicator strain. Transmission electron microscopy (TEM) analysis revealed that KPP105 exhibits typical Sipho-type morphology, characterized by a non-contractile tail and icosahedral head. The bacteriophage features a head approximately 60 nm in diameter and a tail measuring 90 nm in length (Fig. 1A). In plaque assays, KPP105 formed small plaques, approximately 1–2 mm in diameter (Fig. 1B). A one-step growth curve was used to determine its latent period, burst period, and burst size (Fig. 1C). This growth curve exhibited a typical triphasic pattern, with a latent period of about 20 minutes, a burst period from 20 to 60 minutes, and a burst size of 146 PFU per infected cell. Structural proteins of phage KPP105 were analyzed using SDS-PAGE, and five major bands were observed. The molecular weights of these major proteins were compared with those of predicted gene products (PEGs) based on the genome annotation to infer their potential functions. Accordingly, the bands were predicted to correspond to the receptor-binding protein (∼72 kDa), tail fiber proteins (∼41 kDa and ∼33 kDa), tail completion protein (∼28 kDa), and D2 protein (∼26 kDa) (Figure S1A). To assess host specificity, KPP105 infectivity was tested against multiple Gram-negative bacteria, including *K. pneumoniae* (Table 1). Out of the 15 ESBL (+) *K. pneumoniae* strains examined, 12 were susceptible to KPP105, while KBN B-1, K20-KPN-12–057, and K22-KPN-13–007 strains showed resistance. KPP105 did not infect any of the non-*Klebsiella* Gram-negative bacteria tested. These results indicate that KPP105 was capable of infecting a subset of ESBL (+) *K. pneumoniae* strains, while exhibiting no infectivity beyond the *Klebsiella* genus.

**Table 1:** Host specificity determination of isolated bacteriophage KPP105.

Species	Strain	KPP105	
		Spot testing	EOP (%)
ESBL (+) *Klebsiella pneumoniae*	KBN K−1	+	100
	KBN K−2	+	97.6 ± 1.9
	KBN P−1	+	92.3 ± 2.8
	BN B−1	+	0
	K01-KPN−13–134	+	93.3 ± 2.1
	K01-KPN−13–149	+	89.5 ± 3.7
	K07-KPN−13–002	+	75.4 ± 4.3
	K14-KPN−13–016	+	49.5 ± 2.5
	K16-KPN−13–008	+	69.8 ± 1.6
	K16-KPN−13–022	+	75.1 ± 2.5
	K20-KPN−12–057	+	0
	K20-KPN−12–067	+	70.2 ± 3.4
	K21-KPN−12–013	+	82.9 ± 1.9
	K22-KPN−13–007	+	0
	K22-KPN−13–013	+	82.8 ± 2.5
Other Gram-negative bacteria			
*Acinetobacter baumannii*	ATCC17978	-	0
*Citrobacter freundii*	Clinical isolate 15–0628	-	0
*Cronobacter sakazakii*	KCTC2949	-	0
*Escherichia coli*	KCTC1039	-	0
ESBL(+) *E. coli*	K01-ECO12–052	-	0
*Proteus mirabilis*	KCTC2566	-	0
*Pseudomonas aeruginosa*	KCTC2004	-	0
*Salmonella* Typhimurium	ATCC14028	-	0
*Salmonella* Enteritidis	KCCM12021	-	0

+ with lytic activity, - without lytic activity. Efficiency of Plating (EOP) with the host strain KBN K-1 was considered as 100 %.

Given the importance of evaluating stability under various environmental conditions for therapeutic applications, the thermal and pH stability of KPP105 was assessed. The bacteriophage titer was observed to decrease gradually with increasing temperature (Figure S1B). Compared to the initial titer (8.5 ± 0.1 log_10_ PFU/mL), KPP105 remained highly stable at 35 and 40 °C for up to 1 hour. At 50 °C, the phage titer decreased approximately 1,600-fold after 60 minutes (5.3 ± 0.1 log_10_ PFU/mL), although infectious phage particles were still detectable. At 60 °C, a similar level of reduction (∼1,600-fold) was observed within 10 minutes. At 70 °C, the phage was completely inactivated within 10 minutes.

In terms of pH stability, the PFU count (PFU/mL) of KPP105 remained greater than 1.0 × 106, confirming its stability in the pH range of 4–11 (Figure S1C). However, no PFU count was observed at pH 2 and pH 3, indicating that KPP105 is inactive under acidic conditions. The lytic activity of KPP105 at different concentrations was examined by observing bacterial growth over a 5-hour period following phage infection (Figure S1D). The negative control (bacteria without KPP105) exhibited consistent growth, reaching an optical density at 600 nm of about 3.0. In contrast, bacterial growth was inhibited upon phage infection. At a MOI level of 1, bacterial growth was continuously suppressed from the initial infection with KPP105, and at a MOI level of 0.1, growth inhibition was observed starting 1 hour post- infection. These results support the biological properties of KPP105 identified in the one-step growth curve, as the timing of bacterial growth inhibition at a MOI of 0.01 aligns with the latent and burst periods. These findings indicate the effective infectivity of KPP105 and its rapid intracellular replication. In conclusion, KPP105 demonstrated potent infectivity against KBN K-1 strain, efficiently inhibiting bacterial growth even at low MOI levels.

### 3.2 Genomic characterization and phylogenetic analysis of bacteriophage KPP105

**Fig. 2:**
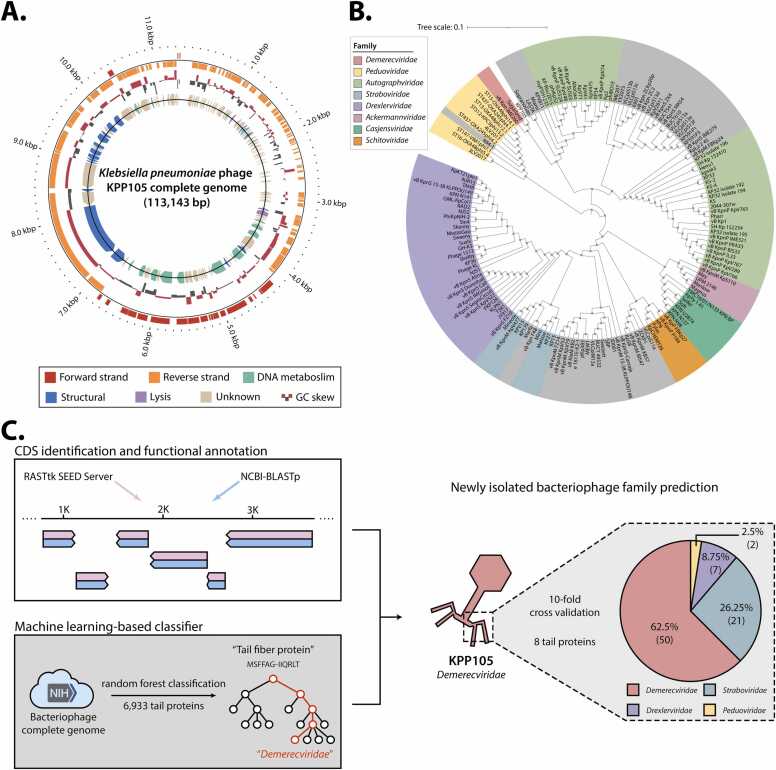
Bioinformatics analysis for the characterization of the isolated bacteriophage. (A) Circular representation of the KPP105 genome. The outermost circles display the organization of contigs for genome annotation, with the red and orange circles representing the forward and reverse strands, respectively. The following inner circles show the GC skew for each genome, and the innermost circle represents functional categorization, including putative lysis gene (purple), putative structural genes (blue), and genes related to DNA metabolism (green). (B) Phylogenetic analysis of 152 *Klebsiella* phages, including KPP105, constructed based on genomic diversity. (C) Schematic of a machine learning model for bacteriophage family classification. A dataset of 6933 tail proteins, obtained from whole-genome sequencing of 714 bacteriophages infecting eight multidrug-resistant pathogenic bacteria, was used. Stratified 10-fold cross validation was performed to reduce training bias during dataset splitting.

The isolated bacteriophage KPP105 was sequenced using both Illumina HiSeq2000 and the PacBIO RSII platform, and bioinformatics-based characterization was performed on the resulting genome sequence data. The genome of KPP105 was assembled into a single linear contig of 113,143 bp, with a coding density of 88.4 % and GC content of 45.6 %. Genome annotation was conducted using the RASTtk pipeline [53], and predicted functions for each open reading frame (ORF) were further confirmed through BLASTp analysis against the NCBI-nr database. The analysis revealed that KPP105 contains a total of 170 ORFs, of which 150 were confirmed as coding sequences (CDSs), and the remaining 20 were predicted to encode tRNAs (Table S1, Fig. 2A). The genome map of KPP105 was extended through comparison with the PHROG database, which enabled the expansion of functional annotation and the evaluation of genomic context to investigate the modularity of the KPP105 (Figure S2). In particular, the modular organization of KPP105 was investigated with a focus on three major functional categories (structural, head and packaging, and lysis-related) commonly observed in bacteriophage genomes. As a result, these modules were found to form well-defined clusters in the KPP105 genome, characterized by gene adjacency and consistent transcriptional orientation. Despite the presence of several hypothetical proteins within these modules, their consistent colocalization with functionally characterized genes supports the structural continuity of the inferred modules. In particular, the lysis module of KPP105 was found to include typical lysis-related genes such as endolysin, holin, O-spanin, and I-spanin (Table S2): PEG.28–29 (endolysin and putative holin), PEG.32–33 (O-spanin and I-spanin), and PEG.69–70 (cell wall hydrolase, metallopeptidase). The genes from PEG.28 to PEG.33 were classified as part of a lysis cassette involved in hydrolyzing of the host bacterial cell wall, based on the typical clustering of bacteriophage lysis cassettes in continuous genetic sequences. Given the structural conservation of functional modules within the genome, the possibility of evolutionary recombination or acquisition of foreign genetic elements was further explored. However, no significant signatures indicative of horizontal gene transfer (HGT) were identified in the genome of KPP105. Furthermore, to expand potential elements associated with the infection strategy of KPP105, additional genome-based screening was conducted based on the expanded functional annotations. As a result, 18 low-confidence anti-CRISPR candidate genes were predicted based on sequence similarity to known Acr proteins, although no self-targeting protospacers were detected (Table S3). Additionally, five genes were annotated under the functional categories of “moron,” “auxiliary metabolic genes,” and “host takeover,” suggesting potential involvement in modulating host physiology.

To explore the evolutionary relationships and genetic diversity of the newly isolated bacteriophage, a pan-genome-based phylogenetic analysis was conducted. This analysis revealed that KPP105 belongs to the *Demerecviridae* family, consistent with the morphological characteristics observed through TEM micrography. Specifically, based on whole-genome alignments against representative genomes from 13 genera within the *Demerecviridae* family (Table S4), KPP105 was further classified into the genus *Sugarlandvirus*, showing the highest average nucleotide identity (ANI) of 94.1 %, in accordance with ICTV taxonomy (Figure S3). Moreover, the pan-genome analysis of KPP105 and its closely related *Demerecviridae* strains identified 126 core genes, 20 accessory genes, and 36 unique genes, which collectively define the *Demerecviridae* clade (Figure S4, Table S5).

The evolutionary relationship of KPP105 was confirmed through phylogenetic analysis, with its morphological features offering valuable insights into its structural characteristics and biological functions. To quantitatively analyze and classify these features, a data-driven machine learning approach, specifically the random forest classification algorithm, was employed [43]. This method was used to accurately evaluate the relationship between morphological features and genomic data, as well as to classify the bacteriophage family based on morphological characteristics derived from amino acid sequences [54], [55], [56]. The classification utilized genomic information from bacteriophage sequences infecting eight major antibiotic-resistant bacteria, including ESKAPE pathogens, sourced from the NCBI RefSeq database. Among the collected data, the *Caudoviricetes* family (95.4 %) was predominant, displaying a wide range of morphological diversity based on tail conformation. Consequently, a total of 714 genome sequences from *Caudoviricetes* were filtered and analyzed (Table S6), and 6933 protein sequences containing the term “Tail” were extracted. These tail proteins were preprocessed and transformed into 431-dimensional features (see Materials and Methods), which were used to train the classifier. The model achieved an overall prediction accuracy of 95.1 % (Figure S5), confirming that KPP105 belongs to the *Demerecviridae* family (Fig. 2C). To further evaluate the generalizability of the classifier, six bacteriophages that had been independently characterized in previous studies [33], [57], [58] and were not included in the training set were tested. Five of them were correctly classified at the family level according to NCBI taxonomy, showing high concordance with existing annotations (Table S7).

### 3.3 Comparative structure-based analysis of receptor-binding protein variability and host specificity in T5-like phages

The physiological and bioinformatics characteristics of KPP105 were examined, leading to the identification of a gene associated with an RBP, PEG.134. Notably, KPP105 is classified within the *Demerecviridae* family, which includes the well-characterized *Escherichia*-infecting phage T5. Previous studies have demonstrated that T5 phage mediates infection by interacting with the outer membrane protein FhuA on the surface of its host, with the structure of this interaction being well-documented (PDB ID: 8A8C) [59]. To explore the relationship between the predicted RBP of KPP105 and its host, further comparative analysis was performed, including sequence analysis, structural prediction, and functional characterization.

**Fig. 3:**
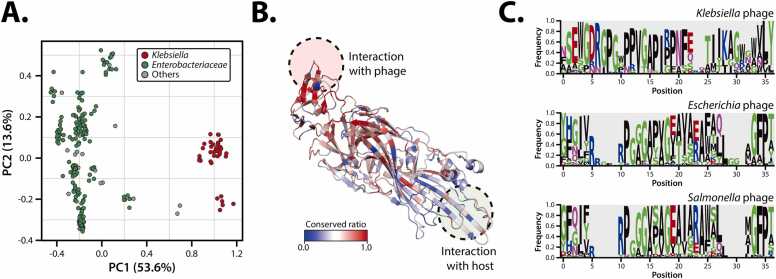
Comparative analysis of structures and sequences of RBPs across different hosts. (A) Principal component analysis of RBPs classified by host type. Phages infecting *Klebsiella* are represented in red, those infecting *Enterobacteriaceae* are in green, and phages infecting other hosts are shown in gray. (B) Structural model of the RBP from KPP105. Conserved regions were depicted using a gradient color scale, where red indicates higher conservation. Conservation was assessed by structural alignment of 268 RBPs. The phage- and host- interaction site is highlighted with red and blue circles, respectively. (C) Sequence logos of the host-interaction regions (positions 219–255) for *Klebsiella*, *Escherichia*, and *Salmonella* phages. These logos represent sequence frequency and host-specific conservation patterns, with blank spaces indicating gaps in the sequence.

BLASTp analysis using the NCBI-nr database identified sequences similar to the KPP105-derived RBP gene. After filtering out unsuitable outliers, 267 homologous sequences were selected for further analysis (Table S8, Figure S7A). These sequences were subsequently used to assess the host spectrum analysis and to perform structural comparisons through a reliable structural prediction pipeline (Figure S6A-B). Sequence-based analysis, utilizing an embedding model (ESM-2) [60] and principal component analysis (PCA), revealed that RBP sequences from *Klebsiella*-infecting phages formed a distinct cluster compared to those from other *Enterobacteriaceae*-infecting phages (Fig. 3A). This clustering pattern was interpreted as reflecting underlying phylogenetic similarity while further providing a basis for subsequent structural comparisons intended to identify host-phage interaction features.

To further investigate the structural features of the host-phage interaction regions, multiple structural alignment (MSTA) was performed based on the predicted protein structures of the collected RBPs (Figure S8). The analysis focused on regions with high alignment ratios, enabling reliable comparison of conservation ratios. Notable differences were observed in conservation between phage- and host-interacting sites, with regions interacting with the phage showing higher conservation ratios than those interacting with the host (Fig. 3B, Figure S7B). Previous structural data on the interaction between T5 phage-derived RBP and host protein FhuA (PDB ID: 8A8C) were used to identify residues within 5 Å proximity. This analysis revealed three major interaction regions containing six or more consecutive residues (129–143, 219–255 and 735–745) (Table S9).

To assess the impact of these identified regions on host specificity, consensus sequences from *Klebsiella*-, *Escherichia*- and *Salmonella*-infecting phages were compared (Fig. 3C). Notably, glycine at position 14, known to play a key role in stabilizing protein-protein interactions, was highly conserved across phages infecting all three genera. In *Klebsiella*-infecting phages, a proline-rich region surrounding position 14 was identified, which may contribute to the strength of protein-protein interactions [61, [62], [63]. In contrast, other *Enterobacteriaceae*-infecting phages predominantly feature amino acids such as glycine, serine, and alanine, which provide relatively higher flexibility, suggesting different binding patterns compared to *Klebsiella*-infecting phages. These findings imply that the proline-rich region in *Klebsiella*-infecting phages and the structural flexibility in other *Enterobacteriaceae*-infecting phages may contribute to distinct host-binding patterns. Additionally, significant differences in amino acid frequency were observed in regions 129–143 and 735–745 between *Klebsiella* and other *Enterobacteriaceae*-infecting phages (Figure S7C), providing critical insights into the determinants of host specificity.

### 3.4 Physiological and structural insights into the lytic mechanisms of bacteriophage KPP105 endolysin with deep learning-based analysis

Physiological characterization of bacteriophage KPP105 revealed key aspects of the lysis process, in which the phage infects host bacteria and induces cell lysis. Genomic analysis identified the presence of lytic cassettes responsible for hydrolyzing the host bacterial cell wall, including PEG.28 (endolysin), PEG.29 (holin), PEG.32 (O-spanin), and PEG.33 (I-spanin). Among these, endolysin was confirmed as an enzyme that directly degrades the PG layer, playing a critical role in the lysis process both structurally and functionally. As a result, structural and functional analysis was performed on the gene predicted to encode the endolysin from KPP105.

**Fig. 4:**
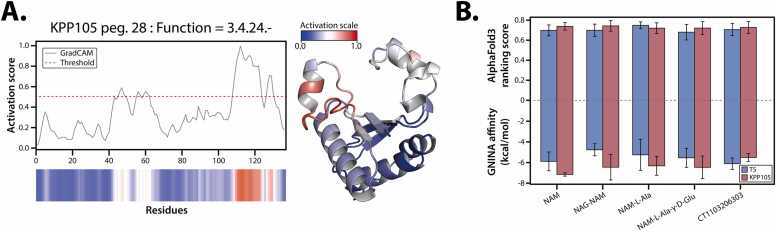
Structural and functional analysis of the putative endolysin derived from KPP105. (A) Functional analysis of the putative endolysin using DeepFRI to identify the EC number and functional residues, with GradCAM applied to assess activation scores. The red dashed line represents the activation threshold (0.5). The 3D structure highlights activated regions, with red indicating higher activation. The predicted structure was generated using AlphaFold3. (B) Affinity analysis of the endolysin with ligands representing various components of the bacterial peptidoglycan cell wall. Five predicted protein-ligand complex structures were evaluated using GNINA to predict binding affinity: NAM (N-acetylmuramic acid), NAG-NAM (dimer of N-acetylglucosamine and N-acetylmuramic acid), NAM-L-Ala (N-acetylmuramoyl-L-alanine), NAM-L-Ala-γ-D-Glu (N-acetylmuramoyl-L-alanine-γ-D-glutamic acid), and CT1103206303 (NAM-L-Ala-γ-D-Glu-L-Lys-D-Ala-D-Ala). The bar graph compares T5- (blue) and KPP105-derived endolysins (red) based on AlphaFold3 ranking scores and GNINA-derived affinities, with error bars representing five replicated models.

Using a reliable protein structure prediction pipeline, AlphaFold3 [28], the structure of KPP105-derived endolysin was predicted, revealing that the protein forms a single globular domain (Figure S6C-D; 2.11 for methodological details). The average ranking score across five models was 92.2, indicating high confidence in the predicted structure. This structure was subsequently analyzed to predict the enzyme EC number, which was then used to identify the activation site for enzymatic activity. EC number prediction, performed using the graph convolutional network-based model DeepFRI, classified KPP105 endolysin as 3.4.24.-, suggesting its function as a metallo-endopeptidase (Fig. 4A). Additionally, the GradCAM analysis was performed to highlight residues critical to enzymatic activity. Regions spanning residues 40–60 and 110–130 were identified as major contributors to hydrolysis, with scores exceeding the threshold of 0.5. These regions correspond to long loop structures previously reported [64] as catalytically significant. Within these regions, conserved residues such as R42, Q46, and S64 were found to match those in T5 endolysin (PDB ID: 2MXZ). Moreover, residues involved in metal ion binding, including H66, D73, and H133, were also found to be fully conserved.

The interactions between KPP105-derived endolysin and various ligands were evaluated to further explore the relationship between binding characteristics and enzymatic activity. Structural modeling was conducted using AlphaFold3, combining KPP105 endolysin with zinc ions and several PG fragments (NAM, NAG-NAM, NAM-L-ALA, NAM-L-ALA- γ-D-GLU, and CT1103206303), with T5 endolysin (PDB ID: 2MXZ) used as a control. Consequently, the predicted structures of KPP105-derived endolysin showed higher ranking scores than T5 endolysin for all ligands except NAM-L-Ala. This suggests that KPP105-derived endolysin may be more structurally compatible with ligand binding compared to T5 endolysin. Additionally, affinity calculations based on the predicted binding structures revealed that KPP105-derived endolysin demonstrated higher stability than T5 endolysin for all ligands, except CT1103206303. Notably, high scores for multiple ligands, including NAM and NAG-NAM, suggest that KPP105-derived endolysin may possess more flexible structural characteristics for ligand binding. Taken together, these findings indicate that KPP105-derived endolysin has a higher ligand binding affinity, providing important rationale for further exploring the relationship between binding characteristics and enzymatic activity.

## 4 Discussion

The growing threat of multidrug-resistant bacteria, particularly *Klebsiella* species, remains a significant global health challenge, emphasizing the urgent need for effective alternative strategies. In this study, we characterized the newly isolated lytic bacteriophage KPP105 through multiple approaches. Specific to *Klebsiella pneumoniae*, KPP105 demonstrates potential for effective use in various physiological environments, showing stability across a broad range of pH levels and temperatures. In addition, the host specificity of KPP105 highlights its ability to target *K. pneumoniae* isolated from clinical environments with high morbidity and mortality rates (Table S10). At the same time, it minimizes disruption to beneficial microbiota. Notably, the host range analysis was conducted using 15 *K. pneumoniae* isolates that were obtained from distinct clinical sources and timepoints, which exhibited diverse antibiotic resistance profiles. This strain-level heterogeneity suggests that the tested panel comprised genetically distinct clonal lineages, rather than closely related variants. This diversity reinforces the significance of KPP105’s infectivity profile. When combined with its morphological features, KPP105 represents a promising candidate for application against clinically significant *K. pneumoniae* strains.

The bioinformatics-based genomic analysis of KPP105 offers crucial insights into the unique characteristics and evolutionary position of this isolated bacteriophage. KPP105 was classified as a strictly lytic bacteriophage based on the absence of lysogeny-associated genes, such as integrase and excisionase, and was further supported by consistent lifestyle predictions across multiple bioinformatics tools [65], [66], [67], [68], [69] (Table S11). In addition, KPP105 was found to possess a complete genome with a high coding density, in which structural, head and packaging, and lysis-related genes were organized into distinct functional modules. In addition, a total of 20 tRNA genes were identified, which may serve to alleviate codon usage bias between the bacteriophage and its host, thereby enhancing translational efficiency and facilitating rapid replication following infection. These features are further supported by morphological observations and one-step growth curve analysis, which revealed a short latent period and a high burst size, suggesting that KPP105 is optimized for a fast and efficient lytic cycle. This lytic strategy is also supported at the molecular level by the presence of a lysis module within the genome. Specifically, genes encoding endolysin, holin, O-spanin, and I-spanin, located consecutively between PEG.28 and PEG.33 and sharing a consistent transcriptional orientation, were interpreted as forming a typical lysis cassette. This genetic configuration may play a role in the effective degradation of the host bacterial cell wall. To further assess the infection strategy of KPP105, the genome was screened for the presence of anti-CRISPR elements and other host interaction factors. As a result, putative anti-CRISPR proteins and host-interaction elements were detected; however, the predictions were of low confidence and lacked supporting evidence such as self-targeting protospacers. Additionally, five genes were annotated under PHROG categories associated with host interaction, although most showed low sequence identity to functionally characterized genes. Therefore, experimental validation will be required to confirm their biological significance. Taken together, KPP105 appears to adopt a “rapid kill” lysis strategy that prioritizes rapid infection and efficient replication rather than relying on alternative infection strategies involving anti-immunity systems or lysogeny-associated elements. To understand the functional characteristics and evolutionary relationships of bacteriophages, it is essential to move beyond single-genome analysis and incorporate phylogenetic approaches based on pan-genome data, which account for patterns of gene conservation and diversity. Through this analysis, KPP105 belongs to the *Demerecviridae* family, sharing a substantial number of core genes that reflect the typical genomic features of this family. This classification contributes to refining the evolutionary placement of KPP105 and provides a basis for clarifying functional mechanisms behind bacterial infection, lysis, and environmental adaptability.

Historically, bacteriophage classification has relied heavily on morphological features observed via electron microscopy, which did not adequately capture genomic diversity and often led to misclassification. In response, the International Committee on Taxonomy of Viruses (ICTV) recently introduced a genome-based, quantitatively structured, and functionally detailed classification system, emphasizing the need for objective and standardized taxonomy [70], [71], [72], [73], [74]. In alignment with this trend, a machine learning-based classification model was developed in this study to classify bacteriophages within the class *Caudoviricetes*, utilizing the sequence-derived physicochemical features of the “Tail” proteins, which are classified as a type of RBPs involved in host specificity [75], [76]. The model was trained using bacteriophages infecting eight different bacterial host species, including members of the ESKAPE group, allowing the model to extract structural sequence features independent of host spectrum. In particular, since the number of available phages infecting *K. pneumoniae* was limited, the training dataset was intentionally constructed to include phages infecting a broader range of hosts. This design was necessary to capture phylogenetic and structural diversity in tail proteins across multiple phage families. As a result, more than 700 phage genomes and thousands of tail protein sequences were incorporated, enabling the model to more robustly learn taxonomically informative patterns. The confusion matrix, which evaluated the performance of the random forest classification model, showed that most bacteriophage families were classified with an accuracy exceeding 0.9. This finding highlights the reliability of tail protein physico-chemical properties for classifying most bacteriophages. In a practical application of the model, the newly isolated bacteriophage KPP105, which was not included in the training dataset, was correctly classified into the family *Demerecviridae*. This classification was found to be consistent with both phylogenetic inference and ICTV-based taxonomy. To further evaluate the generalizability of this model, external validation was conducted using six previously characterized bacteriophages excluded from the training set (Table S7). Among them, five phages were accurately classified in concordance with their current NCBI taxonomic assignments. Moreover, the model was also able to assign a family-level classification to a previously unclassified phage, supporting its robustness and reproducibility. Notably, the model achieved high classification accuracy without relying on dataset bias or algorithmic adjustments, as it was based solely on features directly extracted from protein sequences.

To refine the genus-level classification of KPP105, whole-genome alignments were additionally performed against representative genomes from 13 genera within the *Demerecviridae* family, following current ICTV guidelines [70], [71]. The analysis revealed that KPP105 shared the highest average nucleotide identity (ANI, 94.1 %) with members of the genus *Sugarlandvirus*, supporting its placement within this genus. These results quantitatively complement the machine learning and phylogeny-based classification performed at the family level and are consistent with the genus-level standards recommended by ICTV. Beyond taxonomic classification, pan-genome and phylogenetic analyses were performed to evaluate the gene conservation and uniqueness of KPP105 in relation to other phages within the *Demerecviridae* family. These analyses identified both conserved gene clusters shared with related phages and unique genomic elements specific to KPP105. Such comparative genomic insights are essential for understanding the evolutionary context and potential functional mechanisms of newly isolated phages. Overall, the comprehensive application of machine learning-based classification, ANI-guided genome alignment, and pan-genome and phylogenetic analyses enabled the comprehensive characterization of KPP105 in terms of its taxonomic position and functional genomic features. This framework not only facilitates the accurate classification of novel phages but also provides critical insights into their evolutionary trajectories and infection strategies.

Interpreting bacteriophages within a genomic context is essential for understanding their genetic composition and properties, which in turn helps evaluate their potential applications. However, a more comprehensive understanding of the functional characteristics and host specificity of bacteriophages requires structural analysis at the protein level. In particular, RBPs and endolysins play crucial roles in host infection and the lysis process, and uncovering their structural features provides important insights into the functional mechanisms and potential uses of T5-like phages. Such phages, including those in the *Demerecviridae* family, are known to utilize outer membrane receptor proteins such as FhuA, BtuB, and FepA [77] to facilitate host infection. Since identical receptor proteins are shared across multiple phages or perform similar functions, standardized research is necessary to accurately understand receptor specificity and infection pathways.

Although structural prediction tools like AlphaFold3 have advanced the evaluation of protein-protein interactions (PPIs), the prediction of binding structures for proteins from different organisms, such as pb5-FhuA, has highlighted limitations, including inaccurate interface formation and unreliable ranking scores (Figure S9) [78]. These challenges underscore the difficulties in modeling heterologous protein interactions, particularly in the contexts of antibody and phage research. In this study, structural alignment of RBPs identified three long loop regions involved in receptor binding, with the longest loop found to contribute to diverse interactions with the host receptor. Sequence pattern analysis of this loop revealed differences in amino acid frequencies between *Klebsiella* and *Enterobacteriaceae*. While further investigation is required to determine whether such differences influence host specificity [79], this study provides significant clues for inferring the infectivity of *Klebsiella* and *Enterobacteriaceae* phages.

Additionally, KPP105 contains a single lysis cassette composed of proteins involved in the lysis process, and this study specifically focused on analyzing the structural characteristics of endolysin, which plays a direct role in degrading the cell wall. Endolysins are classified and show host specificity based on the presence of a cell wall-binding domain (CBD) and their mechanism of action on glycosidic and amide bonds [80]. Bioinformatics and structural analysis revealed that the endolysin derived from KPP105 functions as a metallo-endopeptidase, with a single globular domain structure and the absence of a CBD, indicating its specificity toward Gram-negative bacteria. Notably, the deep learning-based analysis identified the functional activation sites of endolysin, suggesting potential for structural engineering. Using diffusion-based AlphaFold3, the binding structures and affinities of endolysin with multiple PG fragments were predicted, with NAM-L-Ala-γ-D-Glu identified as a primary target. This fragment showed a slightly higher ranking score and affinity compared to T5 phage-derived endolysin. These findings provide valuable insights that could inform engineering strategies aimed at enhancing the functionality of endolysin.

In conclusion, this study offers valuable biological insights into the infectivity of *Klebsiella* and *Enterobacteriaceae* through structural analysis of RBP and endolysin and proposes the potential for designing enzymes targeting Gram-negative bacteria. The investigation of endolysin derived from KPP105, combined with deep learning-guided structural analysis, sheds light on the rational design of phage-derived enzymes targeting Gram-negative bacteria. These advancements, which integrate structural insights with computational predictions, along with existing knowledge of RBP-fused endolysins like Innolysins [81], provide a promising platform for expanding or optimizing host spectra, thereby enhancing therapeutic potential against multidrug-resistant pathogens.

## Author statement

All persons who meet authorship criteria are listed as authors, and all authors certify that they have participated sufficiently in the work to take public responsibility for the content, including participation in the concept, design, analysis, writing, or revision of the manuscript. Furthermore, each author certifies that this material or similar material has not been and will not be submitted to or published in any other publication before its appearance in the *Computational and Structural Biotechnology Journal*.

We confirm that the manuscript has been read and approved by all named authors and that there are no other persons who satisfied the criteria for authorship but are not listed. We further confirm that the order of authors listed in the manuscript has been approved by all of us.

The author contributions are provided in accordance with the CRediT guideline. The specific contributions of each author are as follows:

## CRediT authorship contribution statement

**Kim Donghyuk:** Writing – review & editing. **Ko Seyoung:** Writing – original draft, Visualization, Methodology, Data curation, Conceptualization. **Cho Jae-Hyun:** Validation, Investigation. **Kim Jaehyung:** Writing – original draft, Visualization, Methodology, Data curation, Conceptualization. **Kim Youngju:** Validation, Investigation.

## Funding

This work was supported by the National Research Foundation of Korea (NRF) funded by the Ministry of Science and ICT (MSIT) [2022M3A9I5018934].

## Conflict of interest

Y.K, and J-H.C were employed by Optipharm Inc. The remaining authors declare that the research was performed in the absence of any commercial or financial relationships that could be construed as a potential conflict of interest.

## Data Availability

The complete genome sequence and annotation of KPP105 has been deposited to GenBank under the accession number PQ824572.
