# Supplementary material for: Deep learning-guided structural analysis of a novel bacteriophage KPP105 against multidrug-resistant Klebsiella pneumoniae
Source: Comput Struct Biotechnol J. 2025 May 1;27:1827–37. doi: 10.1016/j.csbj.2025.04.032 (PMC12136712; doi:10.1016/j.csbj.2025.04.032)
Supplement: Supplementary file 1 — Supplementary material [file mmc1.docx]

**Supplementary materials**


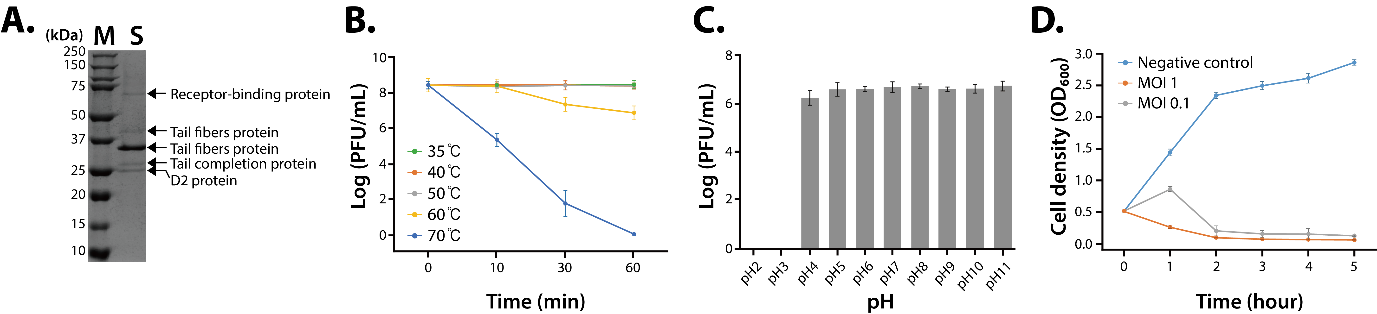


**Supplementary Figure S1. Analysis of protein patterns and biological stability of the isolated bacteriophage KPP105** (A) The protein pattern of KPP105 was analyzed using SDS-PAGE with purified phage particles. Major protein bands were observed at the expected molecular weights. Lane M: protein marker; Lane S: purified KPP105. (B) The evaluation of the effect of temperature on phage titer. The bacteriophage was treated at temperatures ranging from 35 °C to 70 °C for 60 minutes, and thermal stability over time was measured. (C) The analysis of the effect of pH on phage titer. The phage was treated under conditions ranging from pH 2 to pH 11, and titer was subsequently measured. (D) Evaluation of the bactericidal effect of the isolated bacteriophage. The bactericidal effect of the isolated phage was evaluated at different MOI levels (1 and 0.1). Bacteria were infected with KPP105 at MOIs of 1 and 0.1, and cell density (OD_600_) was measured at 1 hour intervals. The negative control consisted of bacteria cultured without bacteriophage.


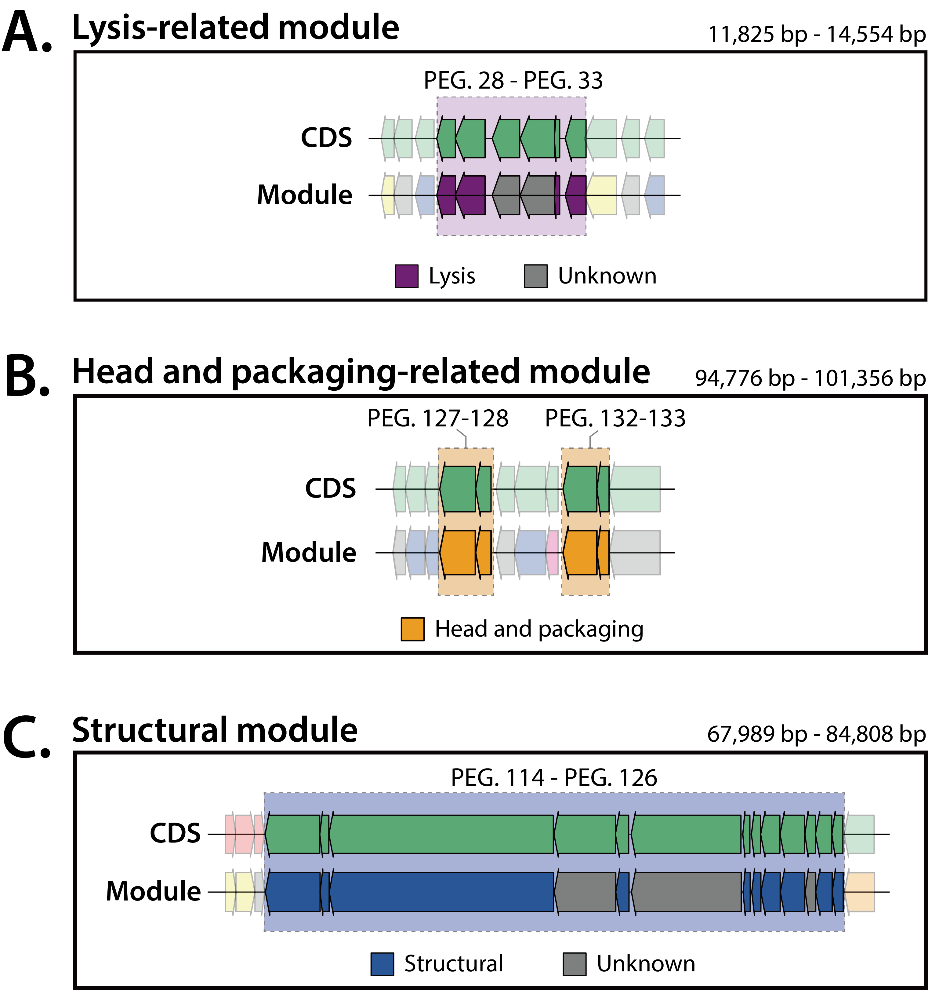


**Supplementary Figure S2. Functional modularity of the bacteriophage KPP105** Schematic representation of the modular genomic organization in bacteriophage KPP105. The modular boundaries were defined based on functional annotation, gene proximity, and consistent transcriptional orientation. Coding sequences (CDSs) are shown as arrows, and functionally coherent modules are highlighted with colored blocks. (A) Lysis-related module (PEG.28–33; 11,825–14,554 bp): includes genes encoding endolysin, holin, O-spanin, and I-spanin (purple), along with adjacent hypothetical proteins (gray), forming a typical lysis cassette. (B) Head and packaging-related module (PEG.127–128, PEG.132–133; 94,776–101,356 bp): spatially separated but functionally linked packaging and head-associated gene blocks (orange). (C) Structural module (PEG.114–126; 67,989–84,808 bp): a contiguous cluster of tail-associated genes (blue), interspersed with hypothetical proteins (gray).


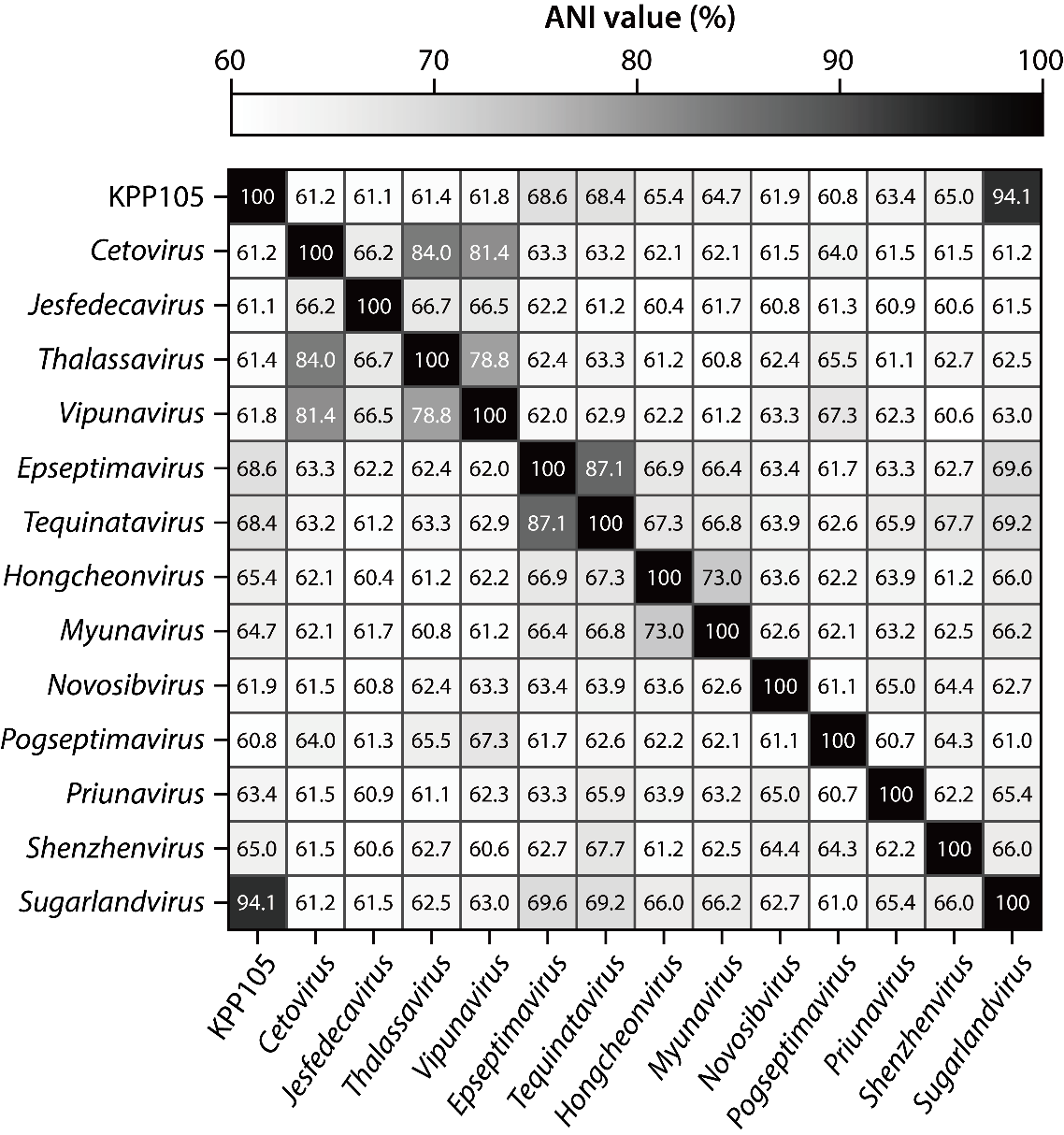


**Supplementary Figure S3. ANI-based genomic similarity between phage KPP105 and representative genomes from 13 genera within the *Demerecviridae* family.** Genomic similarity was assessed between KPP105 and representative genomes from 13 officially recognized genera of the *Demerecviridae* family. KPP105 exhibited the highest similarity (94.1% ANI) with the representative genome of the genus *Sugarlandvirus*. In contrast, ANI values between KPP105 and all other genera were below 70%, indicating distant genomic relationships. Representative genomes for each genus were selected from the NCBI RefSeq database, and ANI values were calculated using the OrthoANI algorithm. Higher ANI values compared to KPP105 are indicated by black, reflecting high genomic similarity.


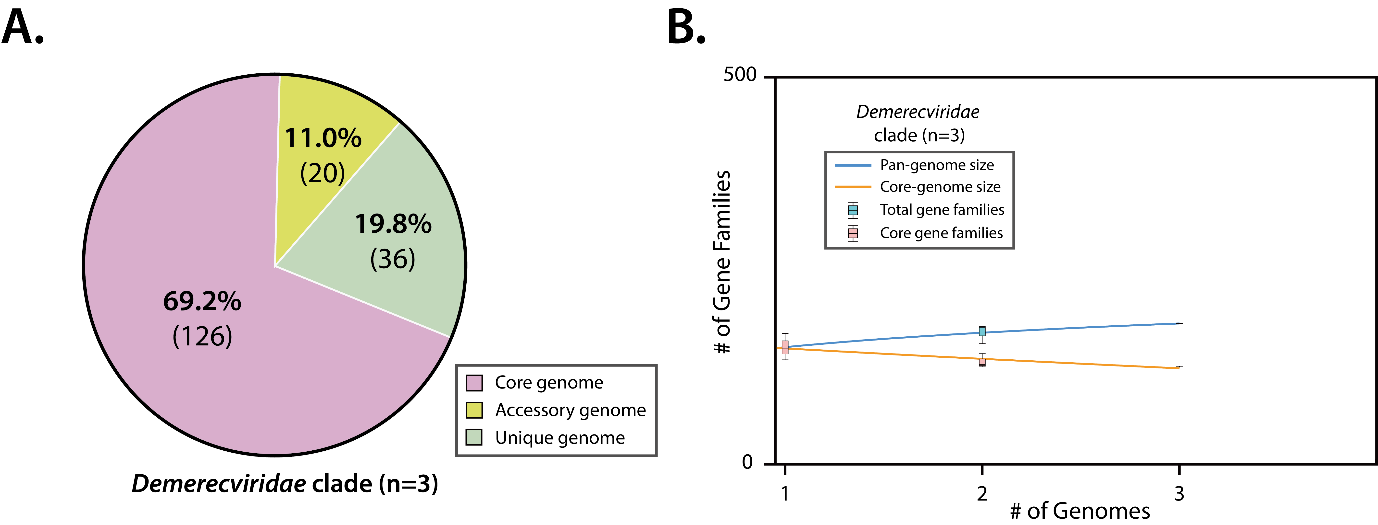


**Supplementary Figure S4. Results of pan-genome analysis of the *Demerecviridae* clade of *Klebsiella* phages based on phylogenetic analysis** (A) Plots represent core, accessory, and unique genes among phages belonging to the *Demerecviridae* clade, respectively. Core genes are defined as gene families present in all analyzed genomes, accessory genes are shared by two or more but not all genomes, and unique genes are found in only one genome. N represents the number of strains. (B) Core-pan genome plot of the *Demerecviridae* clade. The pan-genome analysis emphasizes that the *Demerecviridae* clade of *Klebsiella* phages possesses an open pan-genome structure. The x-axis represents the number of genomes included in the analysis, while the y-axis indicates the number of gene families. The orange line represents the size of the core genome, while the blue line represents the size of the pan-genome.


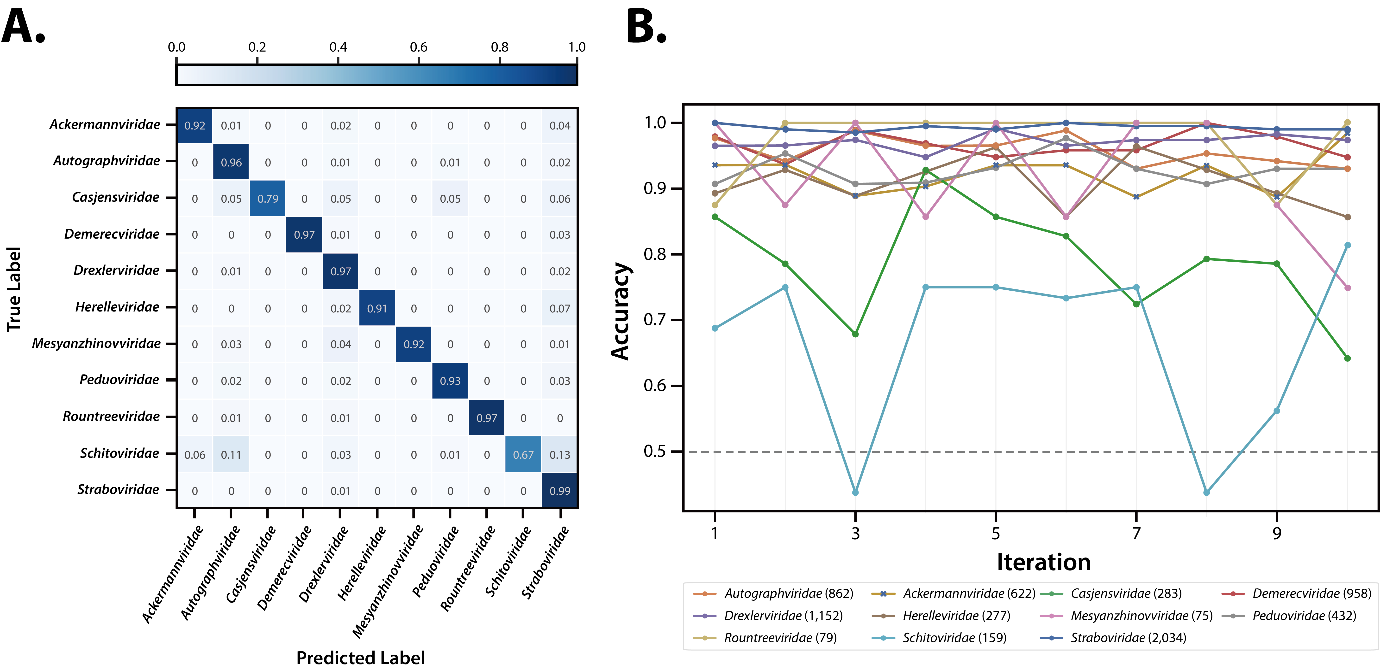


**Supplementary Figure S5. Performance of the machine learning model for bacteriophage family classification** (A) Confusion matrix illustrating the performance of the classification model for 11 bacteriophage family groups. The y-axis represents the true family label instances, while the x-axis represents the predicted label instances. The numbers in each cell indicate the prediction ratio for the true-predicted label pair in the test dataset. (B) Accuracy across iteration during stratified 10-fold cross validation for bacteriophage family groups. The prediction accuracy for each bacteriophage family was evaluated over 10 iterations, with each iteration representing an individual fold in the cross validation process. Bacteriophage family groups are distinguished by different colors.


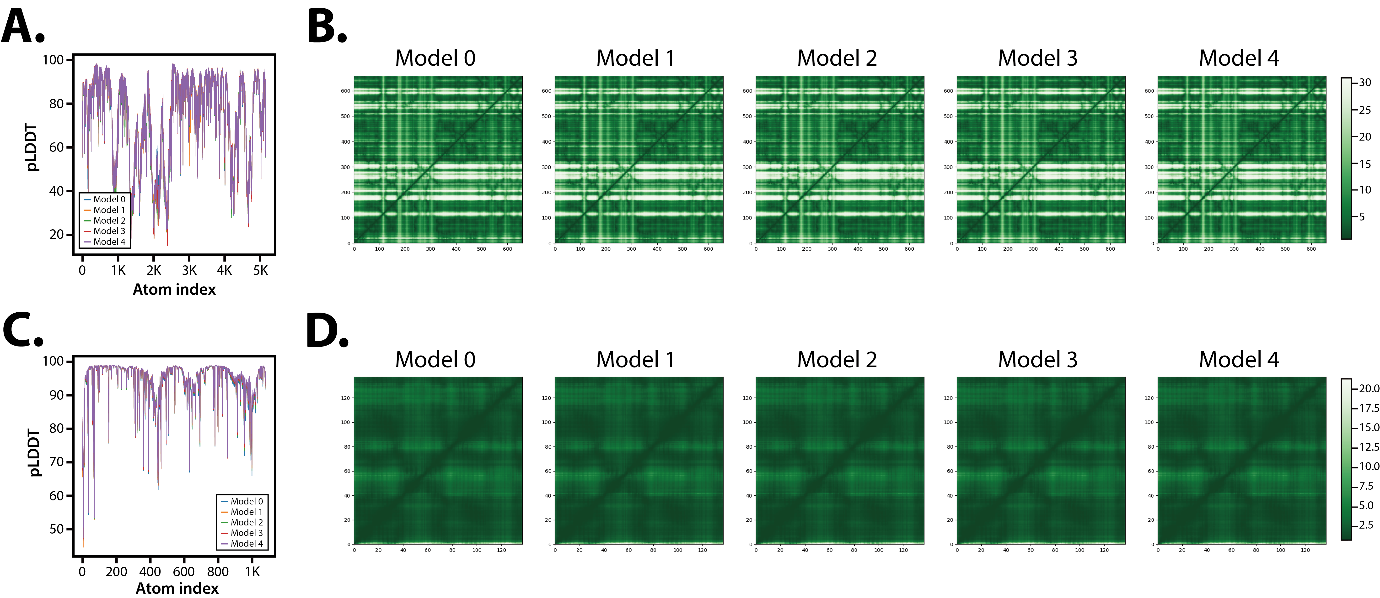


**Supplementary Figure S6. Structural prediction of KPP105-derived proteins using AlphaFold3** AlphaFold3 was employed to predict the structures of KPP105-derived proteins, generating five models (Model 0 to 4). The predicted structures of the receptor-binding protein (A) and putative endolysin (C) derived from KPP105 were arranged based on pLDDT. The x-axis represents the atom indices, and the y-axis represents the pLDDT, with higher values indicating greater prediction confidence in atomic resolution. Additionally, the Predicted Alignment Error (PAE) plots were shown for the receptor-binding protein (B) and endolysin (D) to visualize the confidence in relative domain positions.


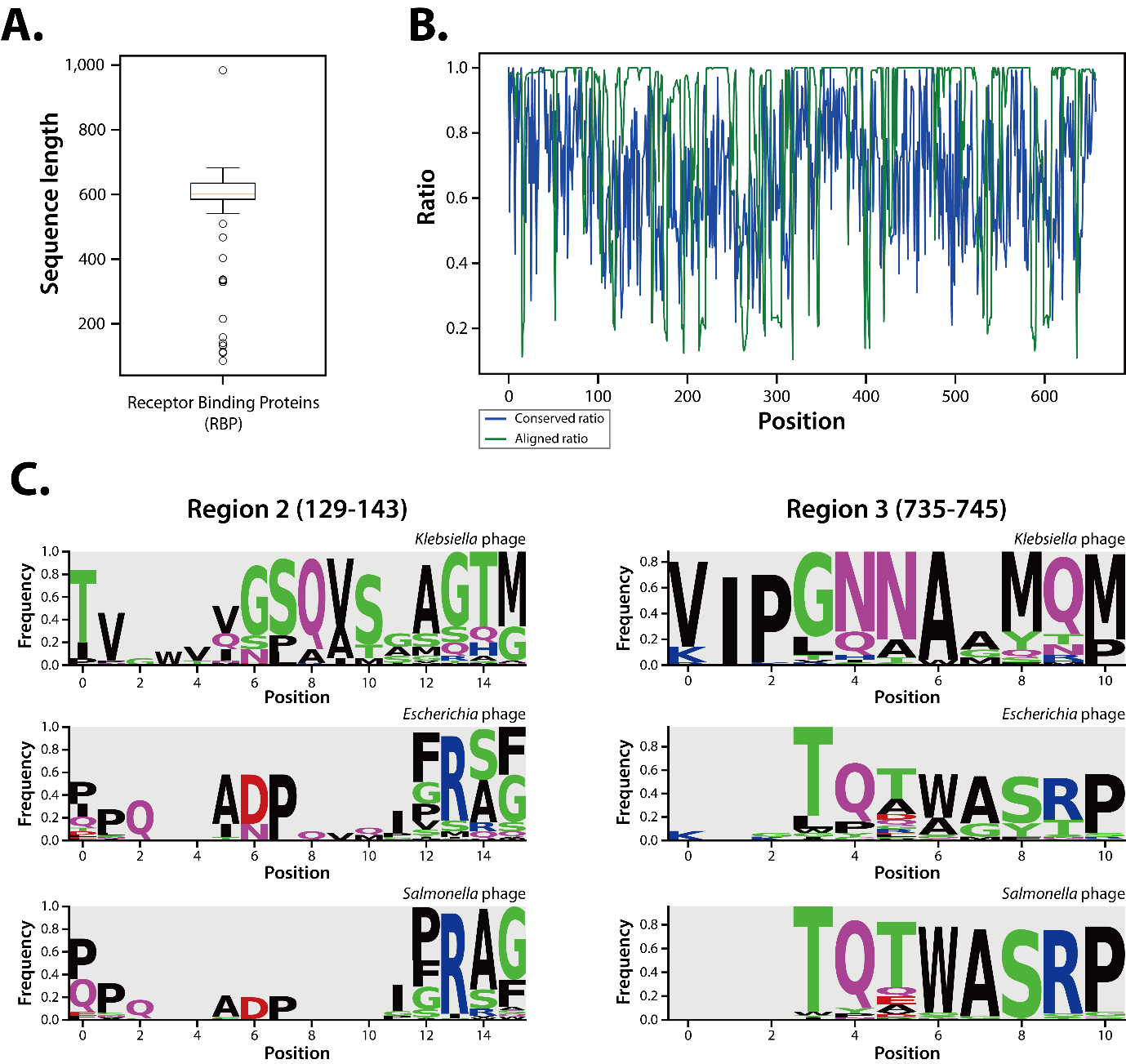


**Supplementary Figure S7. Comparative sequence analysis of host-specific variations in receptor-binding proteins** (A) Sequence length distribution of 282 receptor-binding protein (RBP) hits identified using BLAST from KPP105-derived proteins. The boxplot shows the variability in sequence lengths among the identified hits. (B) Sequence conservation and alignment ratios across the RBPs. The blue line represents the conserved ratio (number of conserved residues per position), and the green line shows the aligned ratio (number of aligned residues per position) across the multiple structural alignment (MSTA) of all identified structures. (C) Frequency distribution of amino acid in two key regions associated with host attachment: Region 2 (positions 129–143) and Region 3 (positions 735–745) in structure-aligned scale. These regions were derived from *Klebsiella*, *Escherichia*, and *Salmonella* phages. The sequence logos highlight host-specific variability in these regions, illustrating that receptor-binding proteins exhibit distinct sequences depending on the host. The gaps observed at some positions in the sequence logos indicate alignment gaps.


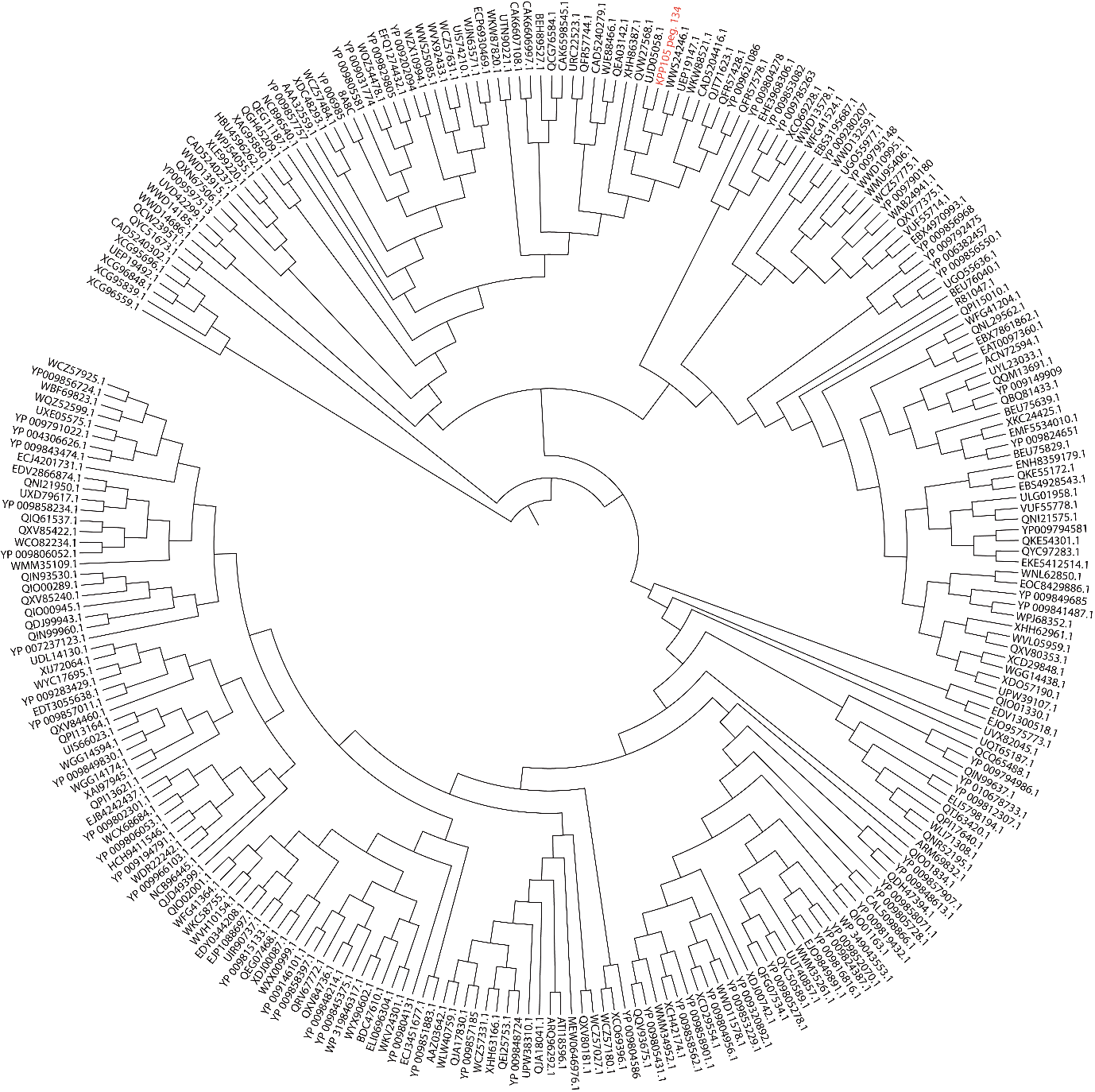


**Supplementary Figure S8. Phylogenetic tree of receptor-binding proteins based on structural alignment** The phylogenetic tree was generated through structural alignment of a total of 268 receptor-binding proteins (RBPs), including the receptor-binding protein derived from KPP105. The structures of all RBPs were predicted using AlphaFold3, with the RBP derived from KPP105 highlighted in red for reference.


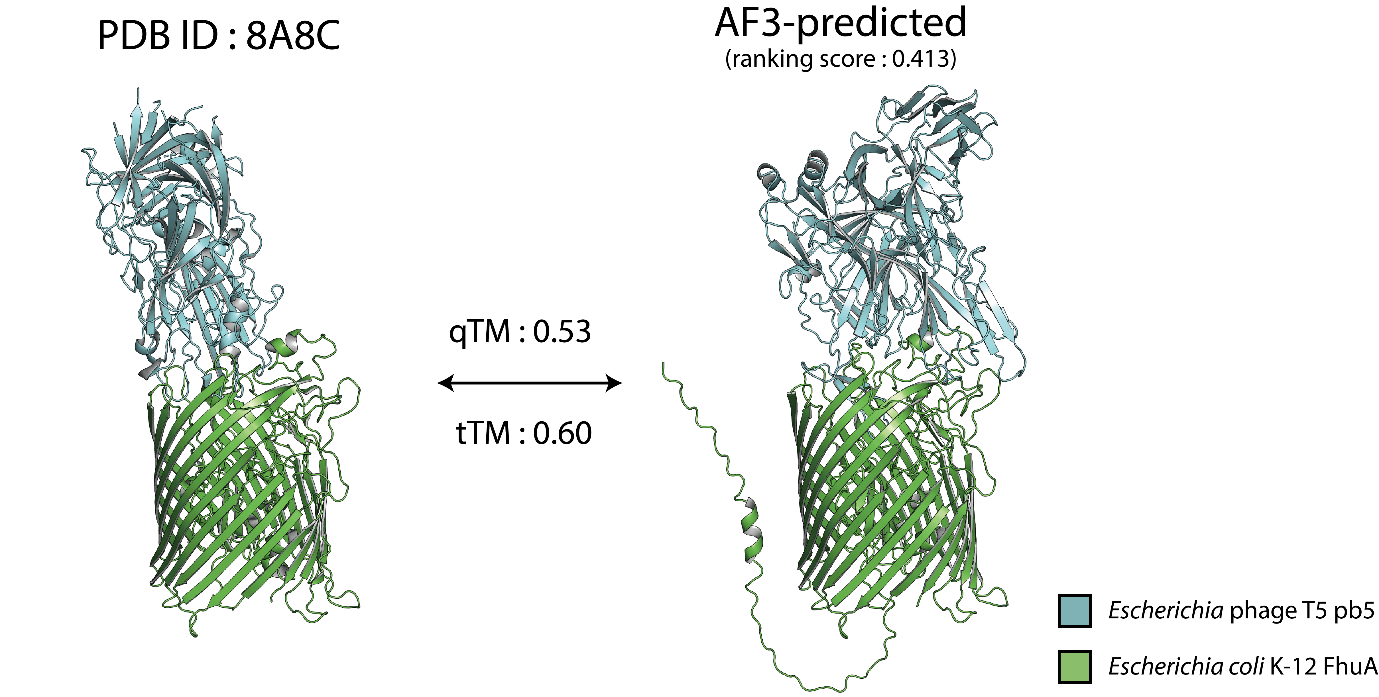


**Supplementary Figure S9. Structural alignment of the predicted structure of the FhuA-pb5 complex based on AlphaFold3 and the crystal structure** The structure prediction of the *E. coli* K-12 FhuA and *Escherichia* T5 phage complex was performed with AlphaFold3, and showed a ranking score of 0.413. The multimer structure alignment was processed with Foldseek-Multimer and compared to PDB: 8A8C.
